# *Mycobacterium tuberculosis* and Pulmonary Rehabilitation: From Novel Pharmacotherapeutic Approaches to Management of Post-Tuberculosis Sequelae

**DOI:** 10.3390/jpm12040569

**Published:** 2022-04-02

**Authors:** Andreea-Daniela Meca, Liliana Mititelu-Tarțău, Maria Bogdan, Lorena Anda Dijmarescu, Ana-Maria Pelin, Liliana Georgeta Foia

**Affiliations:** 1 Department of Pharmacology, Faculty of Pharmacy, University of Medicine and Pharmacy, 200349 Craiova, Romania; andreea_mdc@yahoo.com; 2 Department of Pharmacology, Faculty of Medicine, “Grigore T. Popa” University of Medicine and Pharmacy, 700115 Iasi, Romania; 3 Department of Obstetrics-Gynecology, Faculty of Medicine, University of Medicine and Pharmacy, 200349 Craiova, Romania; lorenadijmarescu@yahoo.com; 4 Department of Pharmaceutical Sciences, Faculty of Medicine and Pharmacy, “Dunarea de Jos” University, 800010 Galati, Romania; anapelin@gmail.com; 5 Department of Biochemistry, Faculty of Medicine, “Grigore T. Popa” University of Medicine and Pharmacy, 700115 Iasi, Romania; lilifoia@yahoo.co.uk

**Keywords:** tuberculosis, antituberculotic drugs, host immune response, pulmonary rehabilitation

## Abstract

Tuberculosis (TB) is still a worldwide public health burden, as more than 1.3 million deaths are expected to be reported in 2021. Even though almost 20 million patients have completed specific anti-TB treatment and survived in 2020, little information is known regarding their pulmonary sequelae, quality of life, and their need to follow rehabilitation services as researchers shifted towards proper diagnosis and treatment rather than analyzing post-disease development. Understanding the underlying immunologic and pathogenic mechanisms during mycobacterial infection, which have been incompletely elucidated until now, and the development of novel anti-TB agents could lead to the proper application of rehabilitation care, as TB sequelae result from interaction between the host and *Mycobacterium tuberculosis*. This review addresses the importance of host immune responses in TB and novel potential anti-TB drugs’ mechanisms, as well as the assessment of risk factors for post-TB disease and usefulness of guidance and optimization of pulmonary rehabilitation. The use of rehabilitation programs for patients who successfully completed anti-tuberculotic treatment represents a potent multifaceted measure in preventing the increase of mortality rates, as researchers conclude that a patient with a TB diagnosis, even when properly completing pharmacotherapy, is threatened by a potential life loss of 4 years, in comparison to healthy individuals. Dissemination of pulmonary rehabilitation services and constant actualization of protocols could strengthen management of post-TB disease among under-resourced individuals.

## 1. Introduction

The extension of rehabilitation programs as constant medical assistance can defy several obstacles in order to increase public health coverage [1]. Nevertheless, it is necessary to integrate these programs in accessible primary healthcare settings, not only in major urbanistic hospitals, for patients to benefit the full potential of rehabilitation [1,2]. Rehabilitation regimens could particularly improve the quality of life for individuals from low- and middle-income countries, taking into consideration that tuberculosis (TB) is the leading cause of death in those areas [2,3]. A holistic approach to TB management could prevent post-treatment complications [4]. The dissemination of rehabilitation services, as well as promoting equity and efficiency of public health measures, could strengthen worldwide health systems’ capacity to ensure the needs of under-resourced populations [5].

The application of rehabilitation programs for patients diagnosed with TB represents a novel multifaceted healthcare service aiming to prevent chronic sequelae, organ failure, and death [6]. At present, there is a lack of protocols regarding pulmonary rehabilitation in TB [7,8,9], although the World Health Organization (WHO) estimated that there were more than 1.3 million deaths in HIV-negative individuals and an additional 214,000 deaths in HIV-positive people in 2020 [10]. An even greater number of deaths and rate of TB incidence is expected in 2021 [10]. Little information is reported regarding the millions of individuals who complete antituberculotic treatment and survive [4,11], more specifically, 19.8 million treated individuals of all ages [10].

In order to properly and equally apply rehabilitation care worldwide, it is imperative to understand the underlying immunologic and pathogenic mechanisms that appear in *Mycobacterium tuberculosis* (*M. tuberculosis*) infection to evaluate the risks of post-antituberculotic treatment complications and to synthesize existing rehabilitation health policies. Moreover, a major public health challenge consists of overcoming the emergence of drug-resistant mycobacterial strains, which can be kept under control through the development of novel anti-TB agents [12,13]. Long-term treatments, as well as mycobacterial survival, often lead to poor adherence, worse outcomes, and pulmonary consequences, even despite a complete pharmacotherapeutic procedure [14,15,16,17]. New therapeutic options and attractive drug targets are currently being analyzed worldwide by researchers and specialists in the field [18,19,20,21,22].

Even more, WHO published a concept note in 2019 recommending that the equity of rehabilitation services could be used as a unique optimization tool for human functioning, the third health indicator among mortality and morbidity [7]. Socioeconomic factors and nutritionally damaging behaviors (such as a poor diet or the absence of physical activity) increase the risk of morbidity in TB endemic regions [2,23,24,25]. A higher incidence of *M. tuberculosis* infection has been recorded in men, chronic smokers, alcohol consumers, and individuals with precarious socioeconomic status [2,10,26]. A delay in TB diagnosis also depends on the patient’s socioeconomic status as it interferes with access to health services. Moreover, time prolongation prior to proper diagnosis directly increases the risk of tissular sequelae [26,27,28]. On the other hand, even though there are millions of patients who are cured and have survived mycobacterial infections, their life expectancy is reduced by four years, according to multiple researchers [23,29,30]. Hoger et al. warns that an average of 3.6 years of potential life loss occurs inpatients upon TB diagnosis, even when properly completing pharmacotherapy, in comparison with healthy humans [31]. Therefore, current re-evaluation of potential targets for novel antituberculotic drugs is crucial.

Even more, after 100 years of BCG (Bacille Calmette-Guerin) vaccine administration, a vaccine which is based on an attenuated strain of *Mycobacterium bovis* [32], more effective strategies are still required to reduce the TB burden [33]. BCG vaccination has proven to grant protection against bacillar dissemination, tuberculous meningitis, and death, rather than reducing the risk of infection, although it is the only vaccine approved until now in TB vaccination schemes [33]. Understanding mycobacterial adaptive and survival pathways in the host environment could lead not only to the development of therapeutic agents, but also to the discovery of novel vaccines [33].

International TB control programs have prioritized screening methods and effective treatment regimens in order to reduce the infection burden on public health systems. Researchers have shifted more towards proper diagnosis and effective treatment rather than understanding post-disease evolution [28,34]. Recovered patients have not been the main focus of intervention programs, although their long-term pulmonary sequelae directly affect their socioeconomic livelihood [35,36,37].

Therefore, the primary objective of this review is to highlight those patients who are not mentioned as often, but who need to benefit from various tools such as rehabilitation in order to improve their quality of life and life expectancy. Post-TB sequelae result from an interaction between the host, the bacillus, and the environment [29,38,39]. Implicitly, it becomes important to understand the specific immune mechanisms that appear during *M. tuberculosis* infection before and after the administration of specific pharmacological agents, in order to select the best rehabilitation program and the patients who would benefit the most.

**Figure 1:**
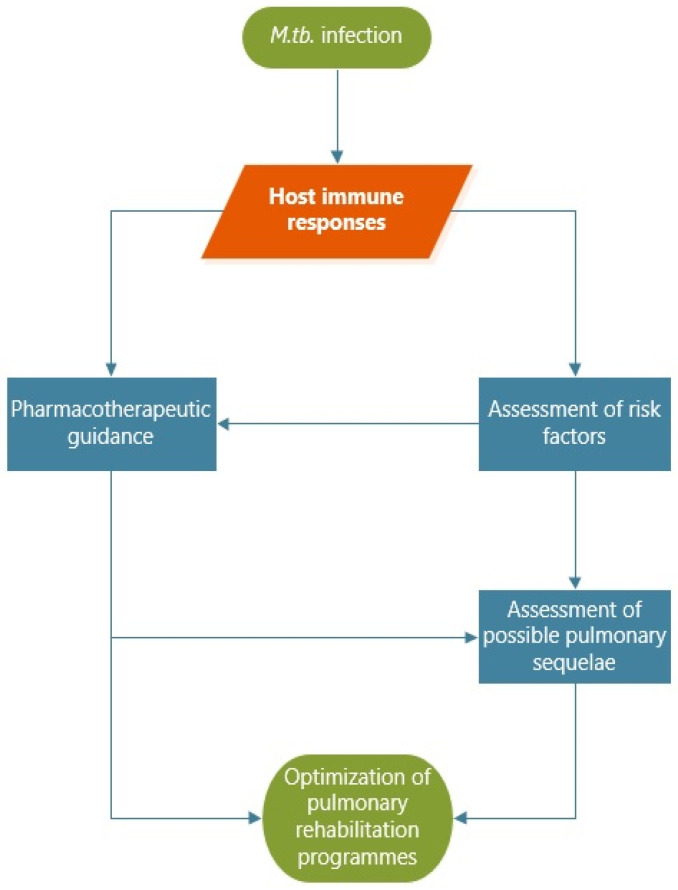
Multidisciplinary study purpose.

This review focuses on (as shown in Figure 1):

- clarifications on the host immune responses in cases of *M. tuberculosis* infection, currently incompletely known;
- guidance on evaluation, future pharmacotherapy, and novel potential antimycobacterial drugs for patients diagnosed with TB after the assessment of risk factors for pulmonary sequelae;
- optimization of pulmonary rehabilitation.

## 2. Pathogenesis and Immune Responses

A study conducted by Jesus and colleagues drew attention to the increased needs and various gaps in physical rehabilitation all over the globe. In 2017, more than 40% of impaired health conditions appeared from a lack of appropriate rehabilitation care [5]. Until now, official rehabilitation guidelines focused mainly upon chronic obstructive pulmonary disease and less on pulmonary TB [9,40]. However, after successful completion of anti-TB treatment, patients may present chronic obstructive respiratory symptoms such as wheezing, cough, sputum production, and dyspnea [3,26]. Recent data has confirmed that chronic lung symptoms among patients who have successfully completed anti-TB treatment increase their death rate and global healthcare burden [30,34].

De Souse Elias Nihues et al. conducted a cross-sectional study in cured TB patients and reported various pulmonary obstructive disorders in almost half of them, following the completion of therapy [26], a result also confirmed by other researchers [41,42]. Visca and colleagues mentioned the higher probability of clinical post-disease consequences from five to six times for patients diagnosed with pulmonary TB in comparison with those diagnosed with latent infections [30]. Based on the study of a cohort of immigrating individuals to Canada from 1985–2015, Basham et al. concluded that more that than 42% of *M. tuberculosis* infected people developed post-disease symptoms in the airways (emphysema, bronchitis, chronic respiratory obstruction) in high resource and low-TB incidence settings, despite the potential availability of pulmonary rehabilitation [43]. The researchers underlined higher social vulnerability due to pulmonary persistent heterogenous sequelae among individuals who successfully completed tuberculostatic treatment [26,27,29] and also reported repeated treatment courses as one of the most important risk factors for post-TB disease [30,44]. Chronic sequelae refer to various obstructive disorders with reduced expiratory capacity, non-responsiveness to bronchodilators, airflow obstruction, bronchiectasis, fibrotic changes, multiple non-tuberculous infections, and aspergillomas that can lead to abnormal spirometry results and impaired diffusing capacity [3,29,45]. Allwood et al. underlined the importance of post-TB lung disease assessment in order to extend life expectancy, although there are still no evidence-based recommendations or guidelines [46,47]. Despite the fact that exacerbations of post-TB pulmonary disease are poorly recognized, symptoms such as hemoptysis may derive from affected and infected parenchyma, pleura, and vasculature [42,45]. The pathogenic patterns of pulmonary post-TB symptoms are difficult to predict [38,42,48]; however, the first innate immune interactions between the bacilli and the human host, although yet poorly understood, are crucial for the outcome of the disease [48,49,50,51].

**Figure 2:**
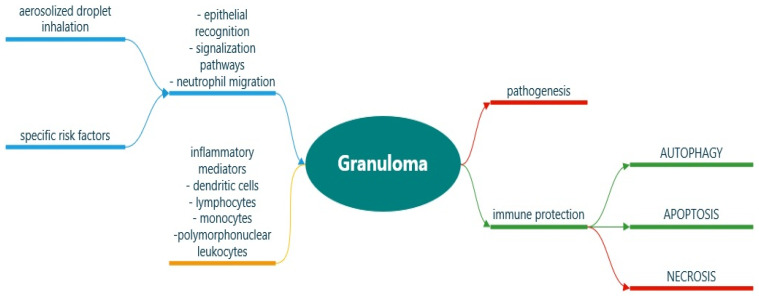
Immunologic pathways in *M. tuberculosis* infection. Color legend: blue and green represent the host innate and adaptive immune responses involved in mycobacterial recognition and removal; red represents mycobacterial survival and long-term tissue inflammation; and orange represents both pathways that can appear during *M. tuberculosis* infection: bacillar death or survival.

*M. tuberculosis* enters pulmonary macrophages after the inhalation of aerosolized droplets and encounters a beneficial long-term survival environment [38,44]. Mycobacteria are intriguing due to their remarkable ability to adapt to the human host after avoiding both the innate and adaptive immune responses [52]. After the epithelial recognition of bacilli (by toll-like receptors), signaling pathways and neutrophil migration are activated, triggering the synthesis of various chemokines and cytokines [49,53,54,55]. Dendritic cells and inflammatory mediators further recruit lymphocytes, monocytes, polymorphonuclear leukocytes, and phagocytes which proliferate and transform into a complex multicellular structure, the so-called histopathological hallmark of TB–granuloma, involved in both pathogenesis and immune protection (as depicted in Figure 2) [44,51,55,56].

During granuloma formation, a protective initial response is observed subsequent to phagocytosis, the host’s attempt to clear the pathogen [47,51]. Alveolar macrophages initiate proinflammatory responses after encountering *M. tuberculosis* in order to restrict its growth, while leucocytes generate pro-oxidative species such as nitric oxide and hydrogen peroxide in balance with antioxidant systems [52]. On the other side, mycobacteria inverts host immune activity through metabolic changes; more specifically, *M. tuberculosis* disrupts the production of NADPH_2_-oxidase (reduced nicotinamide adenine dinucleotide phosphate), leading to granuloma formation, excessive synthesis of reactive oxygen species (ROS), and bacillar replication [47,52]. After bacillary replication, an adaptative immune response is initiated (autophagy), as shown in Figure 2 [47,51,57]. Nevertheless, various antituberculotic agents such as isoniazid and pyrazinamide can induce autophagy during *M. tuberculosis* infection [50,58,59]. Neutrophils are also able to secrete specific antimycobacterial enzymes to support the activity of other immune cells [49,54]. Neutrophils have been recently linked to pulmonary post-TB sequelae after stimulating the pro-inflammatory host response [47,60,61]. The resulted phagosomes represent the host’s attempt at bacillar containment through oxidative burst sustained by neutrophil activity [47,62]. However, the oxidative burst promotes mycobacterial growth by down-regulating the synthesis of protective antioxidants, reducing the T-lymphocytes’ inhibitory activity against *M. tuberculosis*, and by inducing necrosis (unprogrammed accidental cell death) instead of apoptosis (Figure 2) [57,62,63,64]. While apoptosis ensures programmed cellular death without the tissular spilling of cellular contents through nuclear envelope disassembly, cytoskeleton collapse, and inclusion of DNA fragments in specific apoptotic vesicles, necrosis leads to acute inflammation by releasing cellular components into the surrounding tissues [65,66,67,68,69]. *M. tuberculosis* has the ability to generate anti-apoptotic factors that combat specific host pro-apoptotic mechanisms, therefore evading the adaptive immune responses and managing survival [65,66,68]. Necrotic lesions also represent microenvironments for dormant bacilli, which are difficult to target and often resistant to standard pharmacotherapy [70,71,72]. Moreover, the dynamic interactions between the host’s apoptotic immune responses and mycobacterial anti-apoptotic factors decide the outcome of infection [65]. In other words, *M. tuberculosis* disseminates and survives due to its ability to resist apoptosis.

However, Hunter recently argued that pulmonary TB actually begins as a macrophagic infection in individuals with a strong immune response, capable of healing granulomas [73]. The granuloma formation has been considered for many years to be a host protective response, although the mycobacteria manage to evade and to disseminate, even in case of administering proper pharmacotherapy [38,74], undergoing caseous necrosis with early obstructive pulmonary symptoms [73]. The enriched granulomatous center in macrophages which further differentiates into multinucleated giant cells, epithelioid macrophages are the main components of granuloma [51,57]. The immune cells are surrounded by T and B cells able to contain *M. tuberculosis* and prevent bacillar dissemination [49,51,55,57]. Nevertheless, tumor necrosis factor (TNF-α), produced by antigen-presenting cells in the early stages of mycobacterial infection, is essential in granuloma formation [51,75]. On the other hand, granuloma disruption and *M. tuberculosis* dissemination appear in the case of TNF-α blockade (initiated, for example, by anti-rheumatic agents such as adalimumab, infliximab, etanercept, and golimumab) [75,76]. A systematic review conducted by Sartori et al. underlined that the TB incidence in cases of rheumatic patients exposed to TNF-inhibitors was 9.62 per 1000 individuals, with pulmonary TB predominating [76]. Extracellular mycobacterial dissemination appears in cases of macrophage death [55,57,77]. This specific bronchial obstruction leads to macrophagic and lymphocytic dysfunctionalities that will further disrupt *M. tuberculosis* clearance [56,73,74]. Granuloma necrosis can also appear due to a high neutrophil and cytokine inflammatory response [55]. Even more, it seems that a higher cytokine synthesis as an innate immune activity predisposes individuals to an increased probability of a positive tuberculin skin test [49]. Muefong et al. underlined that the neutrophil count in patients with positive sputum-smear test points to a higher bacillary burden and correlates with unfavorable disease outcomes [47].

Although there are current guidelines that specifically recommend appropriate treatment strategies, some individuals develop fibrosis and irreversible tissular modifications [38,47,55]. A cross-sectional study conducted by Ngahane et al. concluded that the presence of fibrotic changes in patients diagnosed with pulmonary TB represents an independent risk factor for future organ impairment [78]. Moreover, the researchers reported lung function impairment in more than 45% of the study participants, despite completion of antituberculotic therapy in all subjects [78]. Calcification and fibrosis associated with a deficit in forced expiratory volume have been associated with increased activity of neutrophils [47,52]. Therefore, development of post-TB pulmonary lesions is related to the persistent host inflammatory responses, even after treatment completion and bacillar clearance [47,79,80]. Guidem et al. concluded that a pulmonary increase of neutrophils, monocytes, and lymphocytes is associated with a higher risk of developing chronic obstructive pulmonary disease (COPD) manifestations in patients who have successfully completed anti-TB treatment [79].

More than 70% of patients diagnosed with TB are malnourished [4,81], and therefore present reduced muscle functionality. Malnutrition also predisposes to unfavorable treatment outcomes and increases death rates among *M. tuberculosis* infected individuals [8,11,82]. Environmental factors such as air pollution, occupational risks, smoking, and alcohol consumption could also lead to unfavorable outcomes after anti-TB therapy due to immunosuppression [23,81]. Nevertheless, cigarette smoke can delay *M. tuberculosis* clearance after cilia paralyze and can interfere with granuloma formation [73]. Additionally, various studies have proven that urban air pollution directly modifies the innate immune response to *M. tuberculosis* infection by altering T-cell functionality and by increasing synthesis of pro-inflammatory cytokines [83,84]. The occurrence of subsequent life-threatening pulmonary infections (especially fungal diseases) after the completion of antituberculotic pharmacotherapy represents a burden among TB survivors, characterized by a slowly-progressive inflammatory response [34,85]. A background of TB is the first risk factor for chronic pulmonary aspergillosis [46,84,85]. Immunocompromised individuals with residual pulmonary cavitation after completion of anti-TB treatment are most likely to express saprophytic colonization and extensive pleural damage [86,87].

Hunter mentions that even though patients may survive after *M. tuberculosis* infection, a body can never recover, as the evolution of the mycobacteria within the host is difficult to predict [73]. A sustainable integrated approach regarding pulmonary rehabilitation plans [2] could improve long-term life quality in prior TB diagnostic and even multi-drug resistant TB (MDR-TB) patients [35,88]. Moreover, recent data confirm that preventing TB sequelae, rather than pharmacotherapeutic strategies, could better influence socioeconomic livelihood [82,88]. However, early TB diagnosis and effective pharmacotherapy are the main preventive methods for post-disease lesions [57,74,89].

Nevertheless, further assessment of rehabilitation programs should be intensively considered and hence, included in research in order to be implemented faster for better management of post-TB treatment patients with pulmonary sequelae. Last, but not least, it is worth mentioning that post-TB survivors may be permanently affected, not only due to pulmonary disease, but also due to other significant organ dysfunctionalities and psychological impact [35,45].

## 3. Pharmacotherapy in Patients Diagnosed with TB

Understanding the underlying immunological mechanisms in TB represents a key in opening the door to anti-TB drug discovery or repurposing pathways. One of the major burdens imposed by *M. tuberculosis* infection is developing novel antituberculotic agents that could further contribute to better outcomes in patients and increased adherence [90,91]. As patients’ compliance increases, the risk of post-TB symptoms reduces [14,28]. This also appears as a worldwide critical demand due to rapid emergence of resistant bacillar strains [91], as no other first-line agent has been approved since the 1960s [92,93], when the combined schema of isoniazid (H), pyrazinamide (Z), rifampicin (R), and ethambutol (E) was completely discovered and introduced into the guidelines [72,90,94]. The minimum duration of first-line standard pharmacotherapy is 6 months, comprised of an intensive phase (HRZE for 2 months) and a continuation phase (HR regimen for 4 months) [14,95]. The first-line treatment targets drug-sensitive mycobacterial strains. Although it usually achieves more than an 80% success rate in cases of newly diagnosed individuals, it can lead to multiple adverse events specific to each active substance (hepatotoxicity, ototoxicity, flu-like syndrome, ocular or nervous toxicity, and much more) [96,97,98].

Although second-line pharmacotherapy is available and recommended to be followed for at least 20 months for patients infected with MDR strains, it has recently been reorganized based upon research regarding drug efficacy and adverse reactions [91,94,99,100,101,102]. The primary agents are clofazimine and linezolid, while p-aminosalicylic acid, one of the first discovered successful anti-TB agents [90], can be introduced as a supplementary drug when needed [91]. Macrolides have proven to have a reduced effectiveness in patients with MDR-TB or extensively drug-resistant (XDR)-TB and have been therefore excluded as second-line drugs [91].

The continuous research from the past years has led to the approval of novel effective anti-TB agents and new mechanisms that could further support lowering the necessity for future rehabilitation programs (Table 1).

### 3.1. Bedaquiline

A lipophilic diarylquinolone called bedaquiline (R207910, TMC-207) was discovered in 2005 through phenotypic screening (a screening process among compound libraries, following antimycobacterial activity against mycobacterial culture cells) and approved in 2012 as a treatment for newly diagnosed patients with MDR-TB [90,92,102]. A total of 109 countries have used bedaquiline as part of their pharmacotherapeutic program for MDR-TB as of the end of 2020 [10]. The major mechanism of action for bedaquiline involves the *M. tuberculosis* proton pump of adenosine triphosphate (ATP) synthesis which subsequently leads to bacillar ATP impairment [92,103]. More specifically, bedaquiline binds with the c subunit of *M. tuberculosis* F_0_F_1_ ATP synthase, preventing the subunit rotation and proton transfer [103,123]. More interestingly, it acts in both replicating and dormant mycobacteria but it does not possess any substantial antimicrobial activity against other bacteria [102,103,107]. Bedaquiline has a risk of prolonging the cardiac QT interval [108,124,125,126]. It is also characterized by a long half-life (more than 150 days) [124,125,126,127]. The association between bedaquiline and other anti-TB drugs (such as fluoroquinolones) which involve risk of QT prolongation is not recommended [109]. Moreover, a significant interaction occurs between R and bedaquiline and their joint use is restricted, as the plasmatic concentration of bedaquiline could be reduced due to CYP3A4 induction [102,127,128]. Currently, phase 1 clinical trials are being conducted in order to identify safer and more potent diarylquinolines compared to bedaquiline, such as TBAJ-876, a 3,5-dialkoxypyridine analogue of bedaquiline, and TBAJ-587, which entered clinical trials in October 2020 [129,130].

### 3.2. Delamanid and Pretomanid

Delamanid (OPC-67683) and pretomanid (PA-824) have been analyzed as potent antituberculotic agents, with both bactericidal and sterilizing activities [130], added in MDR-TB regimens [90,107]. They are nitroimidazoles derivatives which inhibit mycolic acid synthesis (such as keto- and methoxy-mycolic acids [107]) and are able to improve outcomes in MDR-TB patients by affecting both replicating and dormant bacilli [104,105,106]. The mycobacterial cellular wall is crucial for long term survival and its synthesis depends on specific enzymes that are absent in humans. Therefore, it is considered as a potential target for new anti-TB agents [13,123]. Moreover, pretomanid acts as a nitric oxide donor, altering the oxidative mycobacterial balance [108]. Nitric oxide is a molecule which has a key role in the pathogenesis of inflammation. Under normal physiological conditions it shows an anti-inflammatory effect, but under pathological conditions, nitric oxide is considered to be a pro-inflammatory mediator that induces inflammation due to its over-production [131].

Delamanid was approved in 2014 as a treatment for MDR-TB for patients who cannot tolerate second-line regimen [71]. These antibacterial new drugs do not interact with P450 cytochrome and have shown no mutagenicity as of yet, which might minimize interactions with other anti-TB drugs and thus boost their use in individuals co-infected with HIV and *M. tuberculosis* [109,130,132]. However, a transient QTcF prolongation was also confirmed in case of delamanid administration [104], and therefore combination with bedaquiline is not recommended [110]. Nevertheless, an increased risk of cardiac events appears in cases of delamanid or bedaquiline combined with other second-line anti-TB drugs such as clofazimine and fluoroquinolones [110]. The most common claimed adverse reactions of delamanid include gastrointestinal disorders, insomnia, anxiety, tremor, paranesthesia, and migraines [133].

There is limited information regarding their pediatric use or association (trials no. 242-12-232, NCT01859923, NCT01856634) [107,130,132], although delamanid has not proven mutagenicity yet and was approved in 2014 as a potent dose-dependent antituberculotic agent [71,133]. Regarding of its mechanism of action, delamanid can attack residual *M. tuberculosis* from hypoxic and non-hypoxic lesions, as well as necrotizing and non-necrotizing tissues, because it is a prodrug that requires activation by a specific tuberculous deazaflavin (F420)-dependent nitroreductase [71,110,123]. Delamanid seems to be able to decrease fluoroquinolone resistance in mycobacterial strains as well, providing a status of useful associative drug among antituberculotic regimens [109].

The nitroimidazooxazine, pretomanid, has been quite recently approved by the FDA (granted limited population approval in 2019) for patients diagnosed with XDR-TB and intolerant or non-responsive MDR-TB, in combination with bedaquiline and linezolid [130]. Furthermore, pyrazinamide increased both pretomanid and bedaquiline activity when added to the treatment schema [92]. Quadruple therapy consisting of Z, pretomanid, bedaquiline, and moxifloxacin can reduce treatment duration to only three months, in patients diagnosed with MDR-TB [109,110].

### 3.3. Sutezolid and Other Oxazolidinones

Oxazolidinones (such as sutezolid, tedizolid, posizolid, delpazolid, and contezolid [111,112]) have been recently introduced in clinical trials as potent anti-TB drugs due to their inhibitory activity of protein synthesis after binding to the 50s ribosomal subunits [108]. Sutezolid (PNU-100480) and delpazolid (LCB01-0371) are currently in phase 2 clinical trials [111,113,130]. Myelotoxicity is their most important adverse effect besides cytopenia, lactic acidosis, and rhabdomyolysis (data obtained from randomized controlled trial NCT02540460 [113,134]), although sutezolid proved to be a more secure and efficient antituberculosis drug as compared to linezolid, which belongs to the same structural class and is already part of third-line regimens for MDR-TB and XDR-TB [115,135,136]. Another potential adverse event from sutezolid therapy was transient alanine transaminase (ALT) elevations, without life-threatening hepatotoxicity [92]. These adverse events appear to be due to the inhibition of mitochondrial protein synthesis [102]. Linezolid-bedaquiline-pretomanid regimen was approved by the FDA in 2019 [137], although mutations in the 23 rRNA gene seem to be involved in the mechanism of *M. tuberculosis* resistance to linezolid [13,138].

### 3.4. Telacebec (Q203)

Telacebec, a highly lipophilic antitubercular agent, consists of imidazopyridine, which operates independent of cellular oxygen deprivation and mycobacterial replication [90,102,123,139]. Telacebec in nanomolar concentrations restricts *M. tuberculosis* intra- and extra-cellular growth by interfering with ATP synthesis and, implicitly, cellular energy production [108,114]. Its principal target is the respiratory cytochrome *bc*_1_ complex, which is essential for the respiratory electron transport chain involved in ATP synthesis [102,108]. Depletion of mycobacterial ATP leads to cellular death, independent of the replication stage [114,123]. Telacebec was proven to have a 90% oral bioavailability in mice, elevated serum protein binding ability, and a half-life of about 24 h [102]. No interactions with cytochrome P450 were recorded, making telacebec a safe, novel anti-TB drug [102].

### 3.5. Benzothiazinone (BTZ-043) and Macozinone (PBTZ-169, MCZ)

Benzothiazinone is currently advised as a potential antitubercular agent [90]. The primary target for bezothiazinone is the flavoenzyme decaprenyl-phosphoryl-β-d-ribose-20-oxidase (DprE1) [115,117]. DprE1 and DprE2 (decaprenylphosphoryl-2-keto-β-d-erythro-pentose reductase) are essential to the synthesis of arabinogalactan and lipoarabinomannan, main components of the mycobacterial cell wall [117,118]. DprE1 inhibitors block mycobacterial survival by leading to cellular lysis [120,140]. Macozinone is a piperazine derivative with a superior pharmacokinetics profile, security, and pharmacodynamic effect in comparison with the lipophilic benzothiazinone that is less effective in case of severe TB [119]. Moreover, macozinone has proven to have synergistic activity when administered along with bedaquiline and other anti-TB agents [119]. These agents are currently being investigated in phase 2 clinical trials [130]. Another inhibitor of DprE1 is the carbostyril derivate entitled OPC-167832, also currently being evaluated in phase 2 trials [90]. More than 15 compounds have been identified as potent mycobacterial DprE1 inhibitors, including triazoles (377790), nitroquinoxalines (VI-9376), dinitrobenzamides (DNB1), benzothiazoles (TCA1, 7a), carboxy-quinoxalines (Ty38c), thiadiazoles (GSK-710), azaindoles (TBA-7371, currently in phase 1 trials), and pyrazolopyridones [120,140,141,142,143,144].

### 3.6. SQ109

SQ109, a novel small molecule that can be orally administered, is currently being explored in phase 2 trials as a replacement for a first-line anti-TB agent, as it has already proved efficacy against both susceptible and resistant strains [130]. However, SQ109 did not show effectiveness when administered alone [92]. SQ109 (1,2-ethylendiamine derived from the first-line antituberculotic agent ethambutol) has displayed antimycobacterial activity upon ethambutol resistant strains, when administered concomitant with sutezolid and bedaquiline [145,146]. Nevertheless, when combined with standard regimen, SQ109 increased sputum conversion rate by 21% in a prospective randomized double-blind study that included 140 individuals [122]. SQ109 targets Mmpl3 (mycobacterial membrane protein large 3) within the mycobacterial respiratory chain and further manages intrusion in mycobacterial wall synthesis–a unique mechanism among anti-TB agents, as SQ109 is considered a multitarget antituberculotic [121,122]. The Mmpl3 transporter (trehalose mono-mycolate) is essential in mycobacterial wall stability and protein translocation among the membrane, further ensuring pathogenesis [121]. Mmpl3 belongs to a family of export bacterial proteins, but it represents the only protein from the MmpL (mycobacterial membrane protein large) family involved in *M. tuberculosis* survival; therefore, it is a very attractive drug target [120]. In other words, this indolcarboxamide is able to downregulate both the transport of metabolites from mycobacterial cytosol and ATP synthesis [13], with a minimal risk of adverse events (such as gastrointestinal dose-dependent effects) [92]. It could also shorten the average treatment duration [122]. Although SQ109 is structurally derived from ethambutol, it presents poly-pharmacologic properties and multiple bactericidal and antitubercular mechanisms [102]. These are due to the additional ability of SQ109 to inhibit menaquinone and ATP synthesis [102,147]. Both DprE1 and MmpL3 are regarded by researchers as promising antituberculotic drug targets, as several other MmpL3 inhibitors have been reported to have antimycobacterial activity: diarylpyrroles (BM212), adamantyl urea (AU1235), benzimidazoles (C215), indolcarboxamides (NIDT349), dihydrospiro(piperidine-4,4′-thieno(3,2-c)pyrans) (Spiro), tetrahydropyrazolo pyrimidine (THP P), acetamides (E11), piperidinols (PIPD1), and carboxamides (HC2091) [120].

However, it is still difficult to complete the pipeline for anti-TB drug development, as *M. tuberculosis* is a pretentious bacillus that requires environmental facilities and replicates very slowly [13,71]. Joseph and colleagues underlined the importance of the further evaluation and pulmonary care in individuals from their retrospective cohort study, as residual respiratory symptoms (such as chronic cough or breathlessness) were reported in almost 30% of patients although successfully completing first-line standard treatment [93]. Moreover, pathological modifications (cavitation, fibrosis) and hypoxic conditions in patients diagnosed with pulmonary TB may decrease drug bioavailability while allowing *M. tuberculosis* to reside and survive [13,71] and implicitly, to further increase the need of rehabilitation services among patients who may successfully complete pharmacotherapy. On the other hand, the promising activities of novel drugs are not only for their interesting mechanisms, but also for their ability to penetrate thick-walled pulmonary lesions where *M. tuberculosis* resides on long-term in case of telacebec and also for their bactericidal activity in case of MDR and XDR *M. tuberculosis* resistant strains in case of SQ109 [148]. However, are novel anti-TB agents enough for improving the quality of life and decreasing mortality rates in patients diagnosed with pulmonary TB? Matsuo et al. confirm that early interventions of pulmonary rehabilitation are associated with improved human quality of life and survival expectancy [45].

## 4. Pulmonary Rehabilitation

Post-TB sequelae and irreversible extensive pulmonary damage have become top priorities among researchers, as in 2020 more than 150 million *M. tuberculosis* infection survivors have been reported [43,86]. These individuals experienced long-term symptoms associated with aspergillosis, vascular pathologies [87], bronchiectasis, and COPD, in the absence of available pharmacological treatment that could reduce functional pulmonary decline [86,149,150]. The destruction of bronchial wall components during *M. tuberculosis* infection leads to airflow obstruction, bronchogenic spread of purulent sputum, hemoptysis, bronchiectasis, and pneumonia, with consequent symptoms worsening despite completing anti-TB pharmacotherapy [38,151]. Moreover, mixed patterns of ventilatory defects and airflow restrictions (quantified through an increased ratio of FEV_1_/forced vital capacity or a decrease in forced vital capacity) were noted in individuals with TB who further experienced chronic cough, chest pains, and breathlessness [38]. Airflow obstruction appears in these patients due to abnormal healing processes and long-term inflammatory responses such as pleural thickening, bronchovascular distortion, and delimitation of specific fibrotic bands, despite completion of treatment [38].

Daniels et al., in their pilot study, found a decreased exercise capacity and quality of mental and physical life in patients who completed antituberculotic therapy [152]. Gupte et al. obtained abnormal pulmonary functionality in 77% of the patients included in their study, which is regarded as an alarming result after treatment completion [149]. Even more, Gupte et al. showed that only 21% of individuals with post-TB COPD pathogenesis had a beneficial bronchodilator response [149].

Therefore, effective non-pharmacological interventions such as exercise training, behavior management, and patient education are highly necessary [86], due to the lack of guidance regarding the management of post-TB disease [87,149,152]. Pulmonary rehabilitation can be a cost-effective measure, as programs can be held within hospital as well as within the patients’ residence, although supplementary guidance and management of resistance and aerobic training is necessary to be developed for individuals who cannot access pulmonary rehabilitation centers [152].

Lung functionality in patients who completed successfully anti-TB cure can be assessed by performing:

- chest radiography and computed tomography,
- spirometry (including bronchodilator response),
- plethysmography (assessment of lung volumes),
- DLCO (diffusion for carbon oxide),
- arterial blood gas analyses (median arterial blood oxygen saturation and mean arterial oxygen partial pressure),
- evaluation of the capacity to perform exercise via the six minute walk test (6MWT) or the incremental shuttle walk test (ISWT) [4,31,149,150,152,153,154,155].

Radiographic monitoring in patients who completed antituberculotic treatment is useful to predict cavitary infectious diseases, pleural thickening and further colonization with *Aspergillus fumigatus* or other mycobacterial strains [150,156]. Various studies proved that 15% to 25% of patients who completed anti-TB therapy were diagnosed with cavitary aspergilloma [150,156,157]. In other words, management of possible fungal infections in those individuals could lead to higher rates of candidate identification for future pulmonary rehabilitation programs. Moreover, fibrotic patterns, revealed by chest X-rays, can lead to pain or dyspnea (specific symptoms of restrictive ventilatory pathogenesis) [154,158], further selecting post-TB survivors as possible rehabilitation recipients.

Spirometry tests could be used as predictor for post-TB sequelae because a positive response to bronchodilator therapy can prove impaired pulmonary function [149,155,159,160]. Therefore, spirometry monitoring may highlight the actual number of individuals who are in need of pulmonary rehabilitation programs. On the other hand, very recently, Patil and collaborators reported an obstructive pattern after spirometry assessment in 42% of individuals with symptomatic post-TB disease and 32% of individuals without a symptomatic burden after anti-TB treatment completion [161]. Therefore, asymptomatic post-TB survivors may also present defective pulmonary functionality [161,162]. Spirometry analysis is an effective tool in the evaluation of post-TB sequelae and should be included in the identification process of possible candidates for pulmonary rehabilitation, irrespective of symptomatology [161,162]. However, Radovic and collaborators mentioned that spirometry analysis only is not accurate in the detection of possible obstructive pathogenesis and hence, multiple rehabilitation strategies should be approached [163].

Approaching exercise training among patients who survived pulmonary TB requires analysis of patients’ endurance and strength [86]. Several studies reported improvement in patients diagnosed with post-TB pathology after 6MWT and ISWT after measuring forced expiratory volume (FEV_1_), forced vital capacity (FVC), median arterial blood oxygen saturation (SaO_2_), and mean arterial oxygen partial pressure (PaO_2_) [3,27,152]. Lower FEV_1_/FVC ratios are correlated with chronic post-TB airflow obstruction [149,154,160], while lower a FVC result predicts restrictive symptoms [154,158,164]. Approximately 60% of participants from the study conducted by Jones et al. diagnosed with post-TB pathogenesis recorded improvement in the sit-to-stand test and in ISWT, as well as a reduction of restrictive ventilatory symptoms (hemoptysis and pain) [27]. Excessive fibrosis that appears as consequence of tissular healing [158,164] in patients who completed chemotherapy may lead to these restrictive pulmonary disorders [154]. Physical activity is reduced in case of post-TB fungal infections or bronchiectasis, also affecting quality of life [150]. Yang et al. also noticed that obstructive disorders are associated with both reduced quality of life and exercise tolerance, while restrictive ventilatory symptoms lead to lower training ability [154]. In order to limit bronchiectasis clinical symptoms (such as chest pain, respiratory deficiency, fatigue, and cough with hemoptysis), patients should follow rehabilitation programs that include physiotherapy (sputum clearance using hypertonic inhaled solutions) and physical training [150,164].

**Figure 3:**
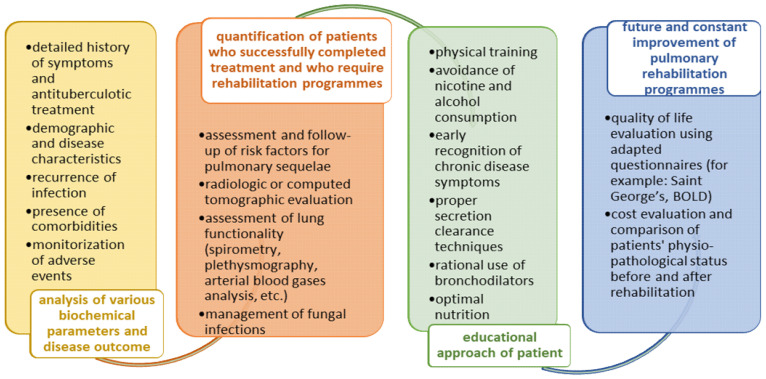
Recommendations for post-TB disease management.

The recovery of muscle function after exercise training in malnourished subjects could also improve absorption of antituberculotic drugs concomitantly with prevention of unfavorable treatment outcomes [8]. Nevertheless, a higher body mass index before antituberculotic treatment onset lowers the risk of lung impairment [149,165]. Yang et al. reported a lesser body mass index as well as a higher rate of nicotine consumption in participants with obstructive ventilatory pathogenesis in comparison to those with normal or restrictive ventilatory symptoms [154]. Singh et al. obtained improvements in dyspnea score, 6MWT and quality of life for TB cured individuals, therefore recommending rehabilitation strategies for core management of post-pulmonary disease sequalae [158]. The recommendations for management of post-TB sequelae are summarized in Figure 3.

Several researchers have recommended nutritional counselling among individuals with post-TB sequelae during rehabilitation programs [4,8], regardless of patients’ age [149]. An impaired quality of life and decreased exercise tolerance are directly correlated with smoking [150,160,166,167]. However, young non-smoking individuals may not be screened for chronic post-TB disease, despite the research conducted by Gupte and collaborators which proved that this population has the highest risk of airflow obstruction development [78,149,166]. Furthermore, a complete pulmonary rehabilitation strategy should include smoking cessation recommendations and avoidance of air pollution [163,167]. Even more, researchers pointed out that irreversible pulmonary damage and various obstructive symptoms appear only if FEV_1_ are lower than 50% [158], so multiple strategies should be followed in order to scale down morbidity and mortality rates in TB survivors [165]. In addition, the complex interactions between *M. tuberculosis* and the host immune response may include various impaired mechanisms in cases of individuals with poor nutritional status, exposed to air pollution or cigarette smoking. Pulmonary rehabilitation may improve host defense strategies by improving exercise ability and strength [27].

Nevertheless, airflow obstruction, excessive pulmonary tissue inflammation and injury, as well as lung functionality decline have been reported in HIV/TB co-infected patients [168,169]. Hoger and colleagues concluded that HIV infected individuals with a history of TB diagnosis were predicted to lose 16 potential years of life [31]. HIV status can therefore predict higher rates of expected life loss in fully treated TB patients [31].

Last, but not least, as we have experienced in the past years a pandemic caused by the severe acute respiratory syndrome coronavirus disease (COVID-19), it is essential to mention those individuals diagnosed with both TB and COVID-19 [170,171]. Although data are extremely limited, in TB patients, symptoms of COVID-19 infection were noted to be more severe and appeared rapidly due to increased host cytokine production, causing a synergistic socioeconomical worldwide burden [170,172,173]. Active TB has also been associated with a 2.1-fold increased risk of developing severe COVID-19; however, more studies with rigorously assessment of bias are necessary [173]. Tadolini et al. underlined that in the group of patients diagnosed with both post-TB sequelae (such as pulmonary infiltrates and cavities) and COVID-19 presented higher rates of mortality [170]. Therefore, it is urgent to gain data from clinical studies in order to predict the impact of this ongoing pandemic on individuals with post-TB disease.

## 5. Conclusions

Despite the constantly increasing efforts over the last years, *M. tuberculosis* infection continues to challenge researchers due to its underlying survival pathways and interactions with the host. The great variability and heterogeneity in pulmonary functionality among individuals who successfully complete anti-TB regimens (ranging from various grades of airflow obstruction and specific lung pathologies such as cavitation, nodular infiltrates, fibrosis, and combination) underlines the multitude of consequences that appear due to the immunologic interaction between the host response and mycobacteria, yet it has been incompletely elucidated. Significant advances have been noted regarding immunological implications and pharmacotherapeutic development, as the more we understand about TB and post-TB sequelae, the sooner novel mycobactericidal mechanisms could be investigated. Moreover, it is also crucial to detect and to quantify patients who require post-disease monitoring, despite completing antituberculotic regimens, as pulmonary symptoms seem to be mediated through host immune responses.

The importance of pulmonary rehabilitation services in individuals who have successfully completed anti-TB treatment has been discussed in this review and a guideline has been proposed. TB control programs and pulmonary rehabilitation services for patients are mandatory, along with the detection of novel, effective, anti-tuberculotic agents and an understanding of mycobacterial mechanisms in order to interrupt the worldwide transmission chain.

## Figures and Tables

**Table 1:** Novel antituberculotic drugs and their mechanisms of action.

Novel Anti-Tuberculotic Drugs	References	Mechanism of Action
**Diarylquinolone** Bedaquiline (R207910, TMC-207)	[92,102,103]	inhibits ATP-synthesis after binding to the c subunit of F_0_F_1_ATP synthase; prevents enzyme rotation and proton transfer within mycobacterial cell; acts on both replicating and dormant bacilli.
**Nitroimidazoles** Delamanid (OPC-67683) Pretomanid (PA-824)	[104,105,106,107] [71,108,109,110]	inhibits mycolic acids synthesis (ketomycolic and methoxymycolic acids) and targets mycobacterial wall; requires activation by a specific deazaflavin F420-dependent nitro-reductase (prodrug); potential decrease in fluoroquinolone resistance; additional activity–nitric oxide donor.
**Oxazolidinones** Sutezolid (PNU-100480) Delpazolid (LCB01-0371)	[111,112,113] [111,112,113]	inhibits mycobacterial protein synthesis; binds to 50 s ribosomal subunits; inhibits mitochondrial protein synthesis (responsible for adverse events such as myelotoxicity).
**Imidazopyridine** Telacebec (Q203)	[90,102,108,114]	inhibits ATP synthesis; binds to respiratory cytochrome *bc*_1_ complex; its activity is independent of mycobacterial replication stage.
**Benzothiazinones** Benzothiazinone (BTZ-043) Macozinone (PBTZ-169, MCZ)	[115,116,117,118] [116,119,120]	DprE1 inhibitors (flavoenzyme decaprenyl-phosphoryl-β-d-ribose-20-oxidase inhibitors); inhibits arabinose synthesis and decreases synthesis of arabinogalactan and lipoarabinomannan (essential components of mycobacterial cellular wall); superior pharmacokinetics and lower risk of adverse events.
**Indolcarboxamide** **(ethambutol derivate)** SQ109	[13,116,119,120,121,122]	multitarget antituberculotic agent; Mmpl3 (Mycobacterial Membrane Protein Large 3)–primary target from respiratory chain; inhibits Mmpl3 transporter (trehalose mono-mycolate) and blocks protein membrane translocation; inhibits ATP synthesis; affects cell wall stability.
